# Overexpression of an *EaZIP* gene devoid of transit peptide sequence induced leaf variegation in tobacco

**DOI:** 10.1371/journal.pone.0175995

**Published:** 2017-04-19

**Authors:** Xiayu Guan, Zhijian Li, Zhiliang Zhang, Xiangying Wei, Jiahua Xie, Jianjun Chen, Qingxi Chen

**Affiliations:** 1 College of Horticulture, Fujian Agriculture and Forestry University, Fuzhou, Fujian, China; 2 University of Florida, IFAS, Department of Environmental Horticulrture and Mid-Florida Research and Education Center, Apopka, Florida, United States of America; 3 Department of Agricultural Water Conservancy, College of Water Resource and Hydropower, Sichuan Agricultural University, Yaan, Sichuan Province, China; 4 Department of Pharmaceutical Sciences, Biomanufacturing Research Institute & Technology Enterprise, North Carolina Central University, Durham, North Carolina, United States of America; United States Department of Agriculture, UNITED STATES

## Abstract

Leaf variegation is an ornamental trait that is not only biologically but also economically important. In our previous study, a Mg-protoporphyrin IX monomethyl ester cyclase homologue, *EaZIP* (*Epipremnum aureum* leucine zipper) was found to be associated with leaf variegation in *Epipremnum aureum* (Linden & Andre) G.S. Bunting. The protein product of this nuclear-encoded gene is targeted back to chloroplast involving in chlorophyll biosynthesis. Based on a web-based homology analysis, the *EaZIP* was found to lack a chloroplast transit peptide (cTP) sequence. In the present study, we tested if overexpression of the *EaZIP* cDNA with or without the cTP sequence could affect leaf variegation. Transgenic tobacco plants overexpressing *EaZIP* genes with (*EaZIPwcTP*) and without (*EaZIPwocTP*) cTP sequence were generated. Many plant lines harboring *EaZIPwocTP* showed variegated leaves, while none of the plant lines with *EaZIPwcTP* produced such a phenotype. Molecular analysis of T0 plants and selfed T1 progeny, as well as observations of tagged marker GFP (green fluorescent protein) did not show any other difference in patterns of gene integrity and expression. Results from this study indicate that transgenic approach for expressing *EaZIPwocTP* could be a novel method of generating variegated plants even through the underlying mechanisms remain to be elucidated.

## Introduction

Leaf variegation is characterized by the presence of discrete patches with different colors in commonly green leaves [1,2]. Since ancient times, consumers and landscape professionals have been relying on the use of variegated plants to increase chromatic diversity, add perceptive layers, and beautify gardens, landscaping surroundings, and interior spaces [3]. To date, ornamental plants with attractive variegated characteristics are holding a significant market share in the worldwide ornamental industry. Leaf variegation is the second most important trait, next to flower color, to the consumers [4].

There are two groups of leaf variegation: pigment-related variegation (chlorophyll and pigment) and structural (air space and epidermis) variegation [5]. Conventional breeding through hybridization is a commonly used method for developing cultivars with different variegation patterns [4]. Novel variegated traits can also be generated using chemical- or radiation-mediated mutagenesis that induces random breakage or rearrangement of DNA sequences [6,7]. Additionally, selection of somaclonal variants from tissue culture derived population is another method of developing variegated cultivars [8–10]. The selected variegated individuals are maintained through vegetative propagation [11]. However, limitations remain on the use of these approaches. Some plants cannot be bred due to the absence of functional reproductive system or certain genetic barriers [12]. Mutation breeding and tissue culture methodologies often lack the expected efficiency and predictability in variegation development [9].

The advent of molecular biology allows for in-depth study of genes and controlling elements responsible for pigment-related variegation and the underlying mechanisms. Thus far, these investigations are primarily conducted using model plant species or staple crops, such as *Arabidopsis*, maize, rice, and tomato [13–15]. Nuclear gene induced variegation mutations account for the majority of the case studies, including *immutans* (*im*), *var1*, and *var2* of *Arabidopsis*, which are capable of conferring variegation phenotypes in Mendelian inheritance and have been extensively characterized [13,14,16]. *IM* controls critical steps in carotenoid biosynthesis but functions in the mitochondrial oxidative pathway [17–19]. Both *VAR1* and *VAR2* genes encode chloroplastic FtsH proteases [20,21]. Mutations in these genes could affect either mitochondria or chloroplasts leading to leaf variegation [14,22–24].

Ornamental plants represent the most abundant and diverse variegated phenotypes in flora of the world. However, studies to unravel molecular regulatory mechanisms using natural variegated ornamental plants remain scarce. In the early 1960s, mutants of poinsettia (*Euphorbia pulcherrima* Willd. Ex Klotzsch) with mosaic patterns of variegation were extensively studied and subsequently linked to segregation of gene mutation following a plastid-based inheritance [25,26]. Our recent study with ‘Golden Pothos’ [*Epipremnum aureum* (Linden & Andre) G.S. Bunting], an important ornamental foliage plant, showed that a reduced level of expression of a nuclear gene *EaZIP* (*Epipremnum aureum* leucine zipper) caused leaf variegation [27]. This gene encodes Mg-protoporphyrin IX monomethyl ester (MgPME) cyclase, which has been found to be associated with plastid development in many organisms ranging from bacteria to higher eukaryotes [28–31]. In plants, this enzyme catalyzes the oxidative cyclization reaction for the formation of an isocyclic ring in divinyl protochlorophyllyllide using the substrate MgPME, thus contributing to the subsequent biogenesis of chlorophylls [32–34]. EaZIP protein shared 84% and 86% identity to *Arabidopsis* (CHL27) and tobacco (NTZIP) orthologs, respectively, indicating the high conservation of this gene among diverse species. In variegated ‘Golden Pothos’, pale yellow leaf tissue showed a more than 4,000-fold reduction in *EaZIP* transcript level compared to the green tissue. Such dramatic down-regulation is correlated with a lower level or even loss of chlorophyll contents in pale yellow tissue and significantly affected otherwise normal chlorophyll biogenesis [27].

In the present study, we conducted extensive homology analysis of *EaZIP* cDNA, which was previously amplified by 5’-RACE PCR [27] with orthologs from other plant species. We found that the previous amplified *EaZIP* does not contain a cTP coding region. Since cTPs in general are responsible for the targeting and translocation of cytosolically synthesized precursors into plastids via post-translational mechanisms [35], the expression of genes encoding chlorophyll biosynthesis enzymes devoid of cTP could affect protein final localization, thereby impairing chlorophyll synthesis and chloroplast development [29, 30]. It might be possible that overexpression of the *EaZIP* cDNA lacking cTP sequence could affect chlorophyll biosynthesis and result in leaf variegation. To test this hypothesis, we used genomic DNA of ‘Golden Pothos’ to amplify an upstream sequence coding region for cTP and created a synthetic *EaZIP* gene with cTP. Transgenic tobacco plants that overexpressed *EaZIP* gene with (*EaZIPwcTP*) or without (*EaZIPwocTP*) cTP were generated and analyzed for their phenotypes. For the first time, we demonstrate herein novel variegated leaves were induced by overexpressing the cTP-truncated gene whereas the cTP-containing gene was unable to produce any phenotypic abnormality. Our study provides a useful tool for efficient engineering of plants with variegated leaves via a gene transfer approach and also presents a valuable system for further investigation of mechanism(s) underlying leaf variegation in ‘Golden Pothos’.

## Materials and methods

### Nucleotide sources, protein sequences and homology analysis

All nucleotide and protein sequences homologous to *EaZIP* of ‘Golden Pothos’ were obtained by searching NCBI GenBank databases (http://www.ncbi.nlm.nih.gov/genbank/). Sequence alignment analysis was conducted by using the Vector NTI Advance^®^ software version 11.5 (InVitrogen, Carlsbad, CA, U.S.A.). A web-based analysis tool ChloroP was used to verify cleavage site position and sequence length of cTP of all EaZIP-related proteins (http://www.cbs.dtu.dk/services/ChloroP/) [36].

### Reversion of the short *EaZIP* using Gibson assembly

To revert the published *EaZIP* short cDNA to one with a putatively full-length cTP coding region, Gibson Assembly reaction (New England Biolabs, Inc., Ipswich, MA, USA) was employed to add a 99-bp upstream DNA fragment to the existing coding sequence. Specifically, a cTP-containing PCR fragment (called fragment A) was generated by using genomic DNA of ‘Golden Pothos’ as template and a primer pair ENEW-51/ENEW-32 that were designed according to 5’ upstream sequences extended from the published cDNA sequence. This extended sequence information was obtained through a promoter cloning effort and verified by similar sequence extension information. The *EaZIP* cDNA coding region was also PCR-modified from the vector CEJ225 [27] as fragment B using a primer pair ENEW-53/ENC-32. The fragment A was 146 bp in length and contained an attB1 recombination site at 5’ terminus followed by a coding region for 33 residues for cTP. The fragment B spans 1,407 bp long with the rest of coding region followed by an attB2 recombination site at the 3’ terminus. Oligonucleotide primers used for PCR reactions were designed by using Vector NTI Advance^®^ software version 11.5 (Invitrogen, Carlsbad, CA, USA) and synthesized by Integrated DNA Technologies Inc. (Coralville, IA, USA). Sequences of these primers were as follows: forward primers ENEW-51 (5’-ggggacaagtttgtacaaaaaagcaggctccgccaccatggcggcggcggcggcggagct-3’) and ENEW-53 (5’-gtccggatgtcctccgcggcgccggc-3’), and reverse primers ENEW-32 (5’- cggaggacatccggaccacgaaggag-3’) and ENC-32 (5’-ggggaccactttgtacaagaaagctgggttcagtaaactagctgtggttcaaaatcagcc-3’).

Gibson assembly reaction requires assembled DNA fragments with an identical overlapped region. There is a 16-bp overlap sequence between fragment A and B. To assemble these two fragments together, PCR products were gel-isolated and used to set up Gibson assembly reaction according to manufacturer’s instruction. The resultant mixture was then subject to gel electrophoresis and DNA with expected fragment size (1,275 bp) was isolated for subsequent cloning to produce pEntry vectors. Since this synthetic gene contains an encoding region for cTP, we named it as *EaZIPwcTP*. For expression analysis, a cDNA sequence complementary to *EaZIP* without cTP region isolated from ‘Golden Pothos’ (GenBank Accession No. FJ666046.1) was re-amplified from the vector CEJ225 [27]. For cloning purpose, PCR primers containing attB1 and attB2 sites including a forward primer ECZ-51 (5’-ggggacaagtttgtacaaaaaagcaggctccgccaccatgtcctccgcggcgccggcgcc-3’) and a reverse primer ENC-32 (5’-ggggaccactttgtacaagaaagctgggttcagtaaactagctgtggttcaaaatcagcc-3’) were designed and used to amplify the *EaZIP* cDNA as a 1,110 bp fragment for subsequent expression analysis. Since this gene does not contain cTP, we named it *EaZIPwocTP*. A sequence homologous to the Kozak fragment ccgccacc was incorporated in upstream region of ATG start codon in the forward primers (ENEW-51 and ECZ-51) of both gene types for efficacious overexpression in test plants.

### Sub-cloning and binary vector construction

For transgene overexpression, a previously described pDestination vector pDRE-2 that utilizes Gateway Clonase technology (Invitrogen) was provided by Dr. Dennis J. Gray of Grape Genetics Laboratory at MREC, UF/IFAS [37]. Both BP and LR Clonase reactions were employed to transfer *EaZIPwcTP* and *EaZIPwocTP* to binary vector according to manufacturer’s instructions (Invitrogen). Specifically, a target gene was first amplified by PCR and primers containing attB1 and attB2 recombination sites at both termini. PCR products were then cloned into a vendor-provided pDONR vector to give rise to pEntry vectors via BP reaction. The pEntry vectors containing the target gene with attL1 and attL2 recombination sites were used for LR reaction with a pDestination vector containing attR1 and attR2 recombination sites to produce pExpression binary vectors. The latter was introduced into *Agrobacterium tumefaciens* strain EHA105 for plant transformation.

Within the T-DNA region of pDRE-2, an EGFP/NPTII fusion marker gene was placed under control of a double enhancer CaMV35S promoter and a CaMV35S transcript termination element upstream of the left border sequence. Immediately following the right border sequence there was a target gene expression unit that was controlled by bidirectional dual promoter complex (BDPC) composed of a double enhancer CaMV35S promoter, a TMV 5’ end untranslated region (Omega element), and a CaMV35S transcript termination element. The latter expression unit was positioned in a divergent orientation relative to the marker gene expression unit [38]. Target gene insertion was carried out by replacing the Cm-R and ccdB containing DNA fragment between attR1 and attR2 sites with attL-containing target gene fragment in pEntry vector via Gateway LR Clonase reaction (Invitrogen, Carlsbad, CA, U.S.A.). Gene sequence in pEntry vector was verified by nucleotide sequencing at ICBR of University of Florida. Up to 4 pEntry vectors containing the same target gene were sequence-analyzed. Only those vectors containing inserts without mutations were then selected for subsequent transfer process. Restriction analysis was employed to further confirm the identity of inserted target genes in pExpression vectors. In addition, a previously described binary vector pd35SG was used as an *EGFP* control [38].

### Tissue culture and plant transformation

Tobacco seeds of (*Nicotiana tabacum* L. cv. Samsun) were surface-sterilized in 20% (v/v) bleach for 10 min followed by a rinse with sterile water three times. Seeds were then plated on hormone-free MS media [39] and cultured under 16-hr light cycle. Transformation of tobacco leaf explants was carried out according to Burrow et al. [39]. Up to 100 leaf disks with 10 disks per culture plate were treated for each vector type. Transformed plant materials were obtained by using kanamycin selection at a concentration of 70 mg/L. Rooted tobacco plantlets were transplanted to 10-cm plastic containers filled with a substrate containing 50% peat, 20% pine bark, 20% perlite, and 10% coarse sand by volume. The plants were acclimatized in a mist bed for a week before being moved to a bench in the greenhouse. When plants reached 15 cm in height they were then moved to 2 gal containers (7.6 L) with the same substrate for further growth to maturity in the greenhouse. For each vector type, 50 to 100 independent plant lines derived from different leaf disks were generated depending on regeneration efficiency. All plants were fertilized with a 15N–7P_2_O_5_–15K_2_O controlled-release fertilizer, Multicote (Haifa Chemicals Ltd, Haifa Bay, Israel) at 15 g per container and watered as needed.

### Molecular analysis of transformed tobacco plants

DNA and RNA were isolated from young leaves of greenhouse-grown plants of tobacco and ‘Golden Pothos’ using extraction kits from Qiagen (Hilden, Germany). To determine the genomic integration of transgenes in transgenic plants, genomic PCR was conducted first using primers targeting either *NPTII* or *EGFP* genes. For *NPTII* gene detection, a forward primer NRT53 (5’-ctgaatgaactgcaggacga-3’) and a reverse primer NRT34 (5’-ctgcttgccgaatatcatgg-3’) were used to amplify a 424 bp fragment from the internal region of *NPTII*. For *EGFP* gene detection, a forward primer EGFP51 (5’-atggtgagcaagggcgaggagctgt-3’) and a reverse primer EGFP32 (5’-cggcatggacgagctgtacaagtaa-3’) were employed to amplify the 720-bp gene. For detection of *EaZIP* sequence in tobacco, a forward primer ENEW-53 (5’-gtccggatgtcctccgcggcgccggc-3’) and a reverse primer ENC-32 (5’-tcagtaaactagctgtggttcaaaatcagcc-3’) was used to amplify an 1145-bp fragment from *EaZIPwcTP* and *EaZIPwocTP*.

To evaluate the copy number of transfer gene, quantitative real-time PCR assay was conducted in a LightCycler 480^®^ instrument equipped with a 96-well plate Therma-base and a software release v1.5 (Roche Molecular Biochemicals, Indianapolis, IN). For such a test, a 20-μl reaction mixture was prepared by mixing 2 μl sample DNA (a total of 6 ng), 10 μl of 2 x SYBR Green I Master PCR buffer (Cat No. 04707516001, Roche), 2 μl of each of primers (0.5 μM) and 4 μl sterile water. Primers include a forward primer EGQ51 (5’-tgaacttcaagatccgccacaacatcgagg-3’) and a reverse primer EGQ32 (5’-aggaccatgtgatcgcgcttctcgttgg-3’). The targeted amplicon measured 172 bp located near the 3’ region of the *EGFP* gene. Samples were replicated three times for each run and experiments were repeated twice. For this test, external controls containing 1 to 4 copies of the *EGFP* gene were used. Such controls were made by diluting E*coRI*-linearized *EGFP* gene-containing plasmid pU203 with a total size of 5,253 bp to a given concentration and by adding the DNA to reaction mixtures that contain 6 ng of non-transformed tobacco DNA. The amount of plasmid DNA corresponding to a single copy of the *EGFP* gene per reaction was calculated as 6 ng (genomic DNA amount) x 5.253*10e3 bp (size of pU203)/4.4*10e9 bp (1C genome size of tobacco, 2n = 4X) = 0.00716 pg/1C. Real-time PCR cycling conditions were as follows: 95°C for 10 min followed by 45 thermal cycles of 95°C for 5 sec, 56°C for 10 sec and 72°C for 40 sec, with a ramping rate of 4.4°C/sec, 2.2°C/sec and 4.4°C/sec, respectively. The level of SYBR-specific fluorescence (483–533 nm) at the end of each cycle was measured and recorded via instrument CCD camera and software. Experiments were repeated twice and results from a single run were presented.

Amplification results were analyzed using instrument software-enabled Absolute Quantification Analysis Using the Second Derivative Maximum Method (Roche) to acquire sample crossing point (Cp) values to extrapolate initial concentration of target DNA in each sample [40]. Transgene copy number equivalent to *EGFP* gene in each transgenic plant was determined based on comparison of Cp-extrapolated target DNA concentrations with in-run standard curve derived from *EGFP* gene-containing plasmid.

For detection of *EaZIP* transcript in transgenic tobacco, a reverse transcription system (A3500) from Promega Co. (Madison, WI, USA) was used. Following the reverse transcription reaction with total RNA, a primer pair EaZIPcDNAF (forward) (5’-acgaaggctaggacgtacac-3’) and EaZIPcDNAR (reverse) (5’-cagagactagtgctgcgatg-3’) was used to amplify a 553 bp fragment near 3’ end region of the gene with above mentioned cycling conditions. PCR products were separated on 0.6% agarose gel via electrophoresis.

### Examination of transgene segregation in progeny

Tobacco seeds are non-endospermic, meaning that the endosperm is absorbed by the embryo resulting in the cotyledons being the predominant tissue in the seed. Seeds from individual tobacco lines were crushed, and GFP expression in cotyledonary tissue was then examined under a dissecting microscope equipped with a fluorescence illuminator and GFP detection filter unit (Leica, Wetzlar, Germany) in order to corroborate transgene segregation in self-pollination-derived progeny. At least 100 seeds were used for the observation. Statistical analysis was used to determine segregation ratios.

### Microscopic observation of chloroplast density in variegated leaves

Leaves of EGFP-CK as a control, *EaZIPwocTP*, and *EaZIPwcTP* tobacco plants at the same growth stage were collected from the greenhouse. Leaf sectors with non-variegated and variegated expressions were excised from whole leaves and their fresh weight in mg was determined, three leaves per treatment samples were ground into slurry using a tissue homogenizer with 500 μl of 0.3M mannitol solution added prior to homogenization. The slurry was then filtered through two layers of sterile cheesecloth. An aliquote (10 μl) of filtrate was loaded onto a hemacytometer and subject to quantification of chloroplast density using a microscope. Results were expressed as the unit of number of chloroplast per mg leaf tissue. Multiple leaf sections were examined and statistical analysis of resulting data was applied.

## Results

### *EaZIP* cDNA and its cTP sequence analysis

Most functional *ZIPs* should produce a peptide with cTP that enabled them to target chloroplasts. However, inspection of the previously published *EaZIP* cDNA sequence from ‘Golden Pothos’ [27] indicated that such cTP was missing. To clarify sequence integrity, we searched NCBI GenBank databases for similar ZIP proteins. Up to 80 such peptide sequences from various species were collected and analyzed for homology by Vector NTI Advance version 11.5. The presence and length of cTP for all peptides were identified using web-based cTP prediction program ChloroP [36]. Among the analyzed ZIP peptides, 73 (89%) peptides contain a cTP with sequence length between 60 to 30 amino acid residues, and 9 (11%) peptides contain no cTP (S1 Fig). The previously published cDNA of *EaZIP* from ‘Golden Pothos’ and also tobacco was among the latter group. In fact, the putative cTP cleavage sites of NTZIP and EaZIP were similar (S1 Fig).

**Fig 1:**
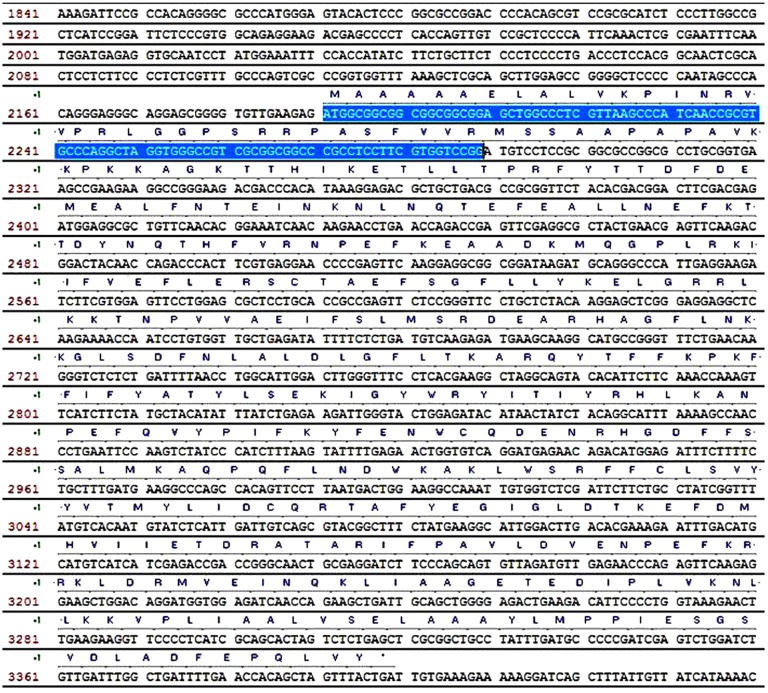
Extended *EaZIP* cDNA sequence of *Epipremnum aureum* ‘Golden Pothos’. Genomic DNA was used for PCR amplification to extend 5’ region of *EaZIP* cDNA. Putative cTP region is highlighted.

To investigate the effects of the cTP, PCR amplification was conducted using genomic DNA of ‘Golden Pothos’ as template and available primer information to amplify an upstream sequence (5’ extension) for cTP. Sequencing analysis of cloned PCR products revealed that there was indeed a translatable region of 99 bp in length from the start codon of the reported cDNA (Fig 1). This extension translation sequence was also analyzed using ChloroP and confirmed to be a true cTP with an extrapolated end at the first start codon of the reported cDNA [27].

A reverted version of the *EaZIP* gene was accordingly constructed using Gibson Assembly method. Sequencing analysis of the 5’ extended region in the reverted gene confirmed 100% identity to pothos sequences recovered from above-mentioned 5’ extension PCR amplification (Fig 1 highlighted region). Both the *EaZIP* short (without cTP) and reverted *EaZIP* (with cTP) genes were subsequently used in expression analysis in transgenic tobacco.

### Recovery of transgenic tobacco plants for overexpression of two versions of *EaZIP*

**Fig 2:**
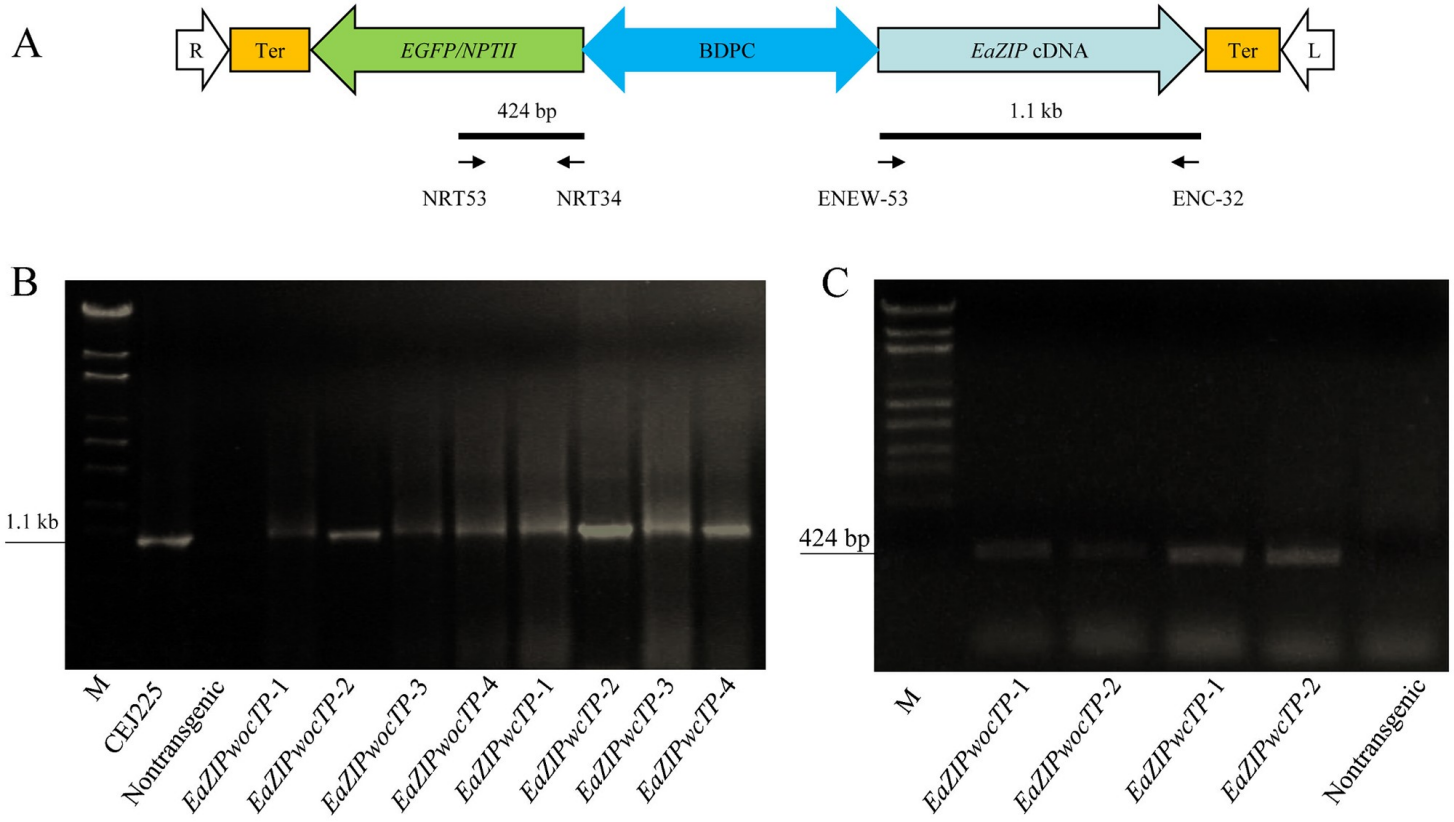
T-DNA and PCR-detection of *EaZIP* and *NPTII* sequences from transgenic tobacco plants. A. Schematic presentation of T-DNA region containing marker gene and *EaZIP* genes (*EaZIPwocTP* or *EaZIPwcTP*) for overexpression analysis. R and L: Right and left borders of T-DNA; EGFP/NPTII: EGFP and NPTII translational fusion marker; BDPC: Bidirectional dual promoter complex derived from double enhancer CaMV35S and CsVMV promoters; Ter: 35S transcript terminator. PCR primers including NRT53, NRT34, ENEW-53 and ENC-32 along with expected amplicons are marked. B. PCR products to show presence of *EaZIP* cDNA fragment in analyzed tobacco lines. M indicates marker DNA, CEJ225: plasmid DNA containing the *EaZIP* short version; nontransgenic control plant; *EaZIPwocTP* lines 1–4; and *EaZIPwcTP* lines 1–4. C. PCR products detecting presence of *NPTII* gene sequence in *EaZIPwocTP* and *EaZIPwcTP* tobacco lines as well as nontransgenic control plant.

Transgenic tobacco plants expressing *EaZIPwcTP* and *EaZIPwocTP* were respectively generated. Both were controlled by a strong divergent promoter complex (Fig 2A), which had been shown to be able to direct constitutive expression of EGFP marker and target genes, simultaneously [37]. Tobacco plant lines transformed with either *EaZIPwcTP* or *EaZIPwocTP* were analyzed by PCR amplification to confirm gene integration. Plasmid DNA CEJ225 [27] was used as a positive control for *EaZIP* while genomic DNA from a non-transformed tobacco plant was used as a negative control. As shown in Fig 2B with four representive lines from each genetic construct, PCR analysis confirmed that all tested plants harbored the expected 1.1 kb amplicon identical to that amplified from the positive control CEJ225. Non-transformed tobacco did not produce any PCR product. The absence of 1.1 kb cDNA-derived *EaZIP* sequence in control tobacco could be due to the fact that DNA sequence of endogenous ZIP gene(s) of tobacco, such as AY221168 has highly divergent sequence compared to *EaZIP* at both forward and reverse primer designing sites, thus does not give rise to amplicons with expected size. The sequentially linked *NPTII* gene in the form of an *EGFP-NPTII* fusion to facilitate the recovery of transgenic plants and monitor gene expression status was also detected by the gene specific pair of primers to amplify a 424-bp fragment [37] in some transgenic plants (Fig 2C). In correlation with findings from DNA analysis by PCR detection, normal GFP expression was also observed in all regenerated individual plants after kanamycin selection (data not shown). These observations indicate that both transgenes were integrated into the genome of transgenic tobacco lines and the inserted genes were successfully expressed.

### Copy number determination and phenotypic segregation analysis

Seven transgenic tobacco plants along with a non-transformed plant were subject to quantitative real-time PCR to determine copy numbers of genome-integrated *EGFP*. In agreement with regular PCR analysis, no signal was detected from non-transformed control tobacco plant (Table 1). Among four analyzed transgenic plants containing the *EaZIPwocTP*, two harbor a single copy, whereas the other two contain three copies. From three tested plant lines with the *EaZIPwcTP*, one plant each was found to contain one, two, and three copies, respectively.

**Table 1:** Determination of transgene *EGFP* copy numbers using quantitative real-time PCR based on external plasmid controls.

Sample	Mean Cp^w^	STD Cp^x^	Mean conc.^y^	STD conc.
Vector-CK1^z^	23.676	0.132	1.0	0.207
Vector-CK2	22.816	0.069	2.4	0.146
Vector-CK3	22.668	0.167	3.0	0.325
Vector-CK4	21.790	0.026	4.0	0.002
Nontransgenic plant	31.564	0.228	0.0	0.000
*EaZIPwocTP*-1	22.601	0.059	3.2	0.113
*EaZIPwocTP*-2	24.054	0.063	0.6	0.061
*EaZIPwocTP*-3	22.791	0.000	2.8	0.001
*EaZIPwocTP*-4	23.254	0.137	1.0	0.004
*EaZIPwcTP*-2	23.340	0.138	1.0	0.003
*EaZIPwcTP*-5	23.298	0.074	1.7	0.146
*EaZIPwcTP*-10	22.755	0.016	2.8	0.034

^w^ Average values of crossing point from three sample replicates

^x^ Standard deviations of average crossing point values

^y^ Average estimates of copy number extrapolated from Mean Cp values

^z^ Copy number standards reconstructed using quantified plasmid DNA

**Fig 3:**
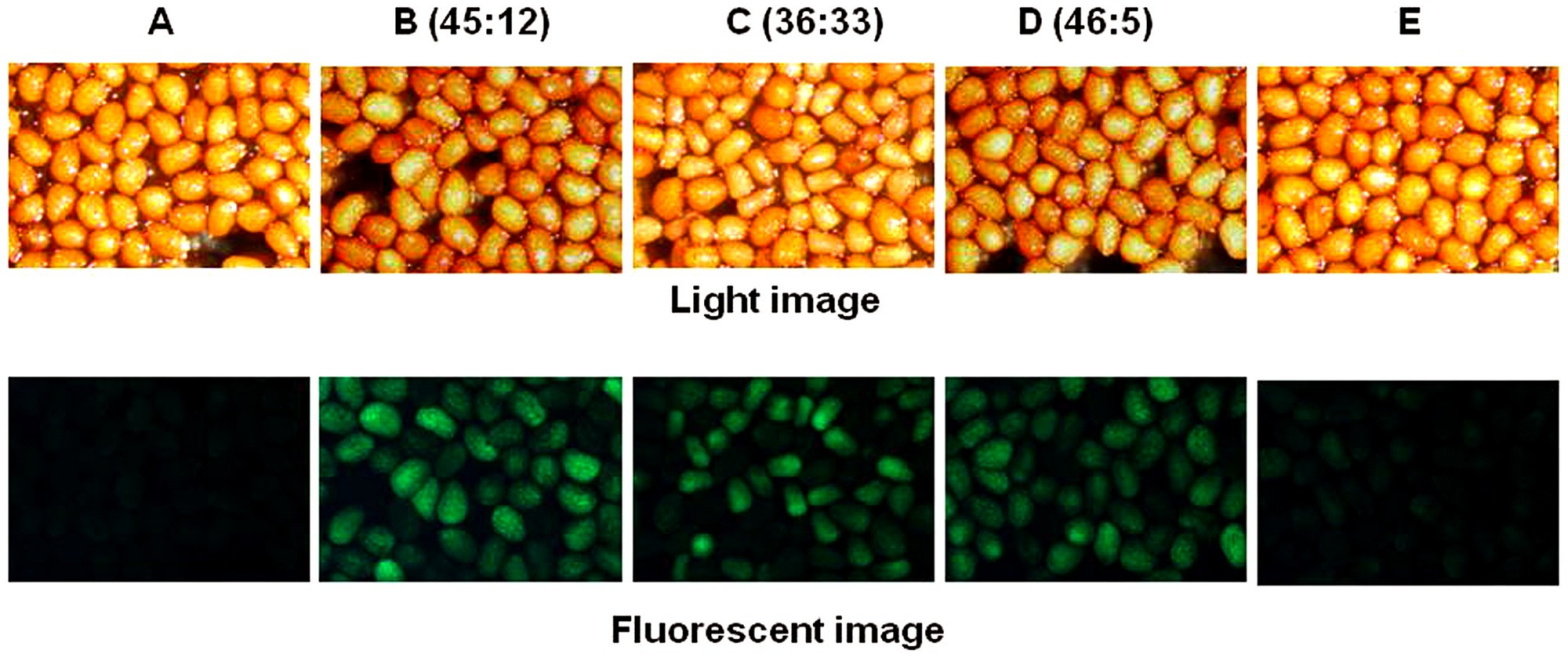
GFP expression segregation in seeds of tobacco T1 progeny overexpressing *EaZIP*. Tobacco plants were grown to maturity. Seeds were collected from each line and subject to GFP expression observation. Representative images from examined lines were presented. Segregation ratios between GFP positive and negative seeds were indicated in parentheses. A, non-transformed tobacco plant; B and C: *EaZIPwocTP* plant lines; and D and E: *EaZIPwcTP* plant lines.

EGFP expression was also monitored in seeds of selfed progeny of four transgenic tobacco lines (Fig 3). GFP was not detected in non-transformed tobacco plants (Fig 3A). A number of plant lines with single copy transgene indeed showed 3:1 segregation (Fig 3B). However, as described previously by Yin et al [41], some lines displayed odd ratios of GFP positive and negative segregation such as 1:1 or even 10:1 (Fig 3C and 3D). Some lines produced seeds with extremely low levels of GFP expression (Fig 3E). This non-Mendelian inheritance of transgene may be associated with polyploidy genetic background of tobacco and its interaction with introduced transgene [41]. There seems no clear correlation of segregation ratios among examined plants with the type of *EaZIP* genes introduced. Another possibility could be due to the silence of transgene; this phenomenon has been shown quite common in transgenic plants [42].

### *EaZIP* transcription in transgenic tobacco lines

**Fig 4:**
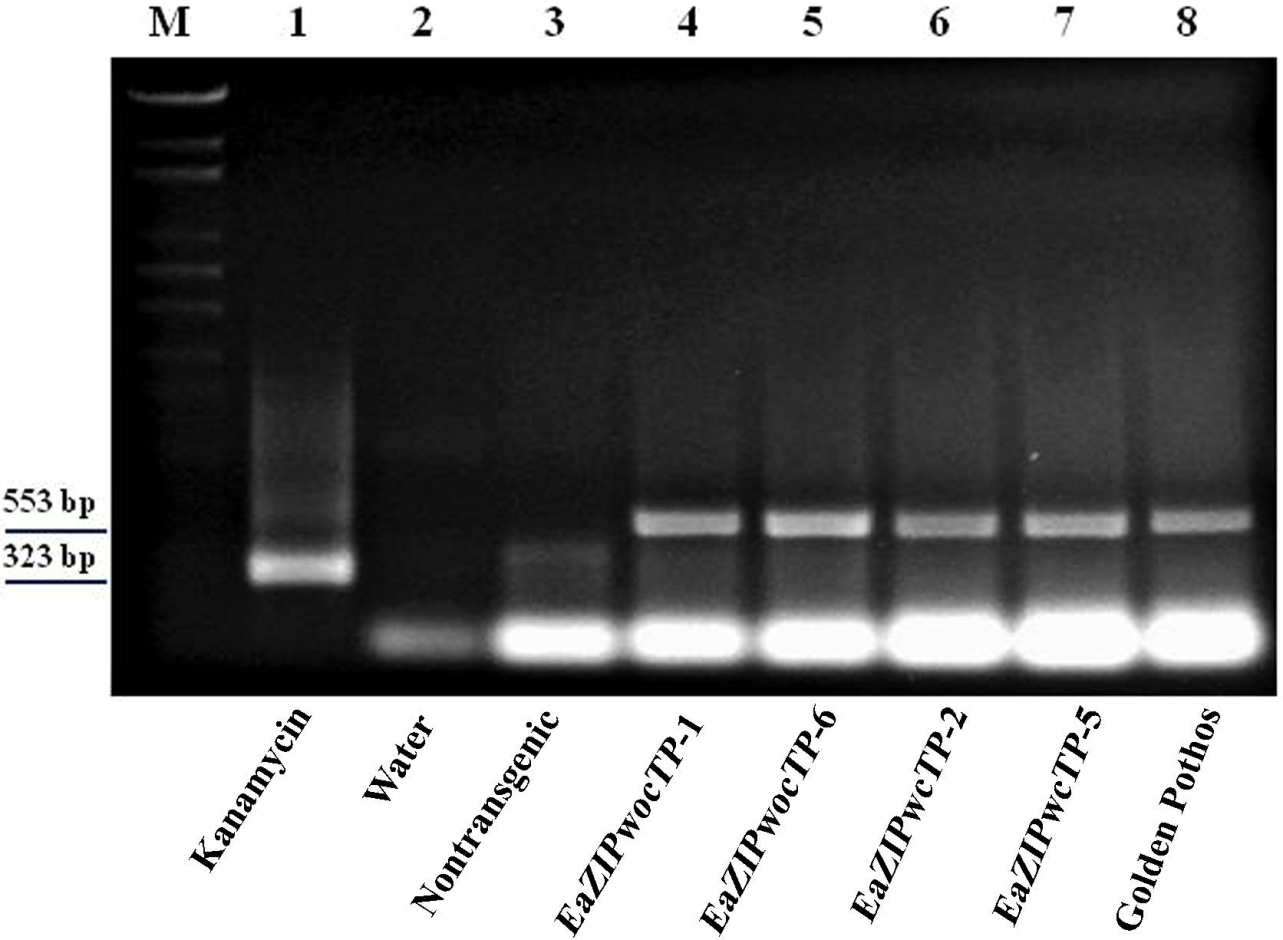
Detection of *EaZIP* transcripts from tobacco and pothos plants. Total RNA was isolated from leaf tissues of indicated tobacco and pothos plants and subject to reverse transcription reaction followed by amplification of target fragments using regular PCR. M indicates marker DNA. Kanamycin gene amplicon is 323 bp in length, and *EaZIP* gene amplicon is 553 bp in length.

To determine if the introduced *EaZIP* gene was expressed, reverse transcription followed by amplification of *EaZIP*-specific cDNA was carried out. As shown in Fig 4, a positive control derived from a kanamycin resistance gene provided by vendor was successfully amplified as a 323 bp (lane 1). A negative control composed of water did not generate expected amplification signal (lane 2). Likewise, RNA sample from a non-transformed tobacco plant also did not produce expected amplicon (lane 3). On the other hand, the expected 553 bp fragment was amplified from samples of all four randomly selected *EaZIP*-expressing tobacco plants (lanes 4 to 7). This unique product was identical to that detected from RNA sample from ‘Golden Pothos’ (lane 8). Repeated amplification of cDNA using alternative *EaZIP*-specific primers for longer amplicon also resulted in identical results (data not shown). These results suggest that the *EaZIP* gene introduced into a tobacco was faithfully transcribed.

### Phenotype of tobacco plants containing *EaZIP* genes

Transgenic tobacco plant lines were obtained after stringent selection and regeneration process using kanamycin. Up to 20 and 65 plant lines overexpressing *EaZIPwocTP* and *EaZIPwcTP* were respectively recovered and successfully acclimatized in a greenhouse along with non-transformed plants and transformed harboring p35SG vector.

**Fig 5:**
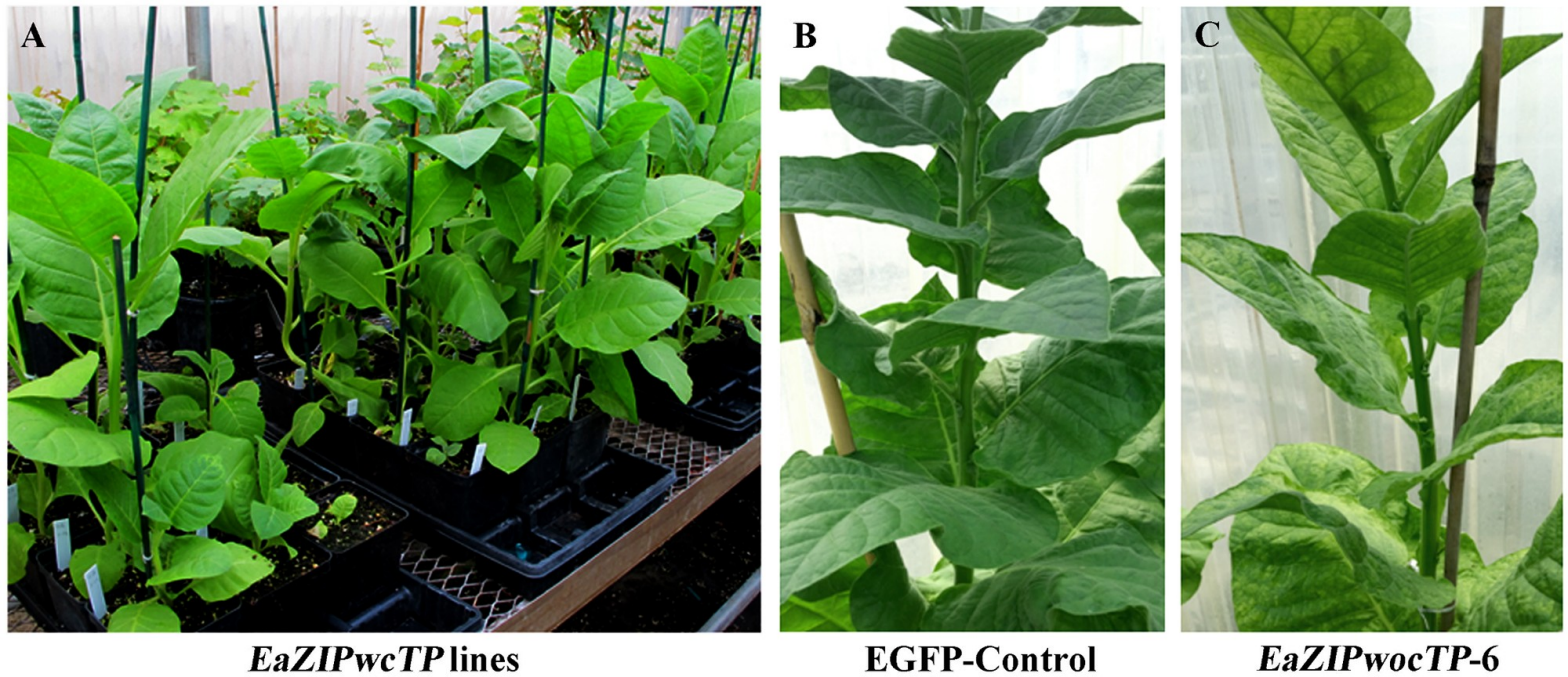
Transgenic tobacco plants grown in a greenhouse. A. independent lines expressing *EaZIPwcTP* (no variegated leaves); B. an *EGFP* line (no variegated leaves); and C. an *EaZIPwocTP* line with variegated leaves.

**Fig 6:**
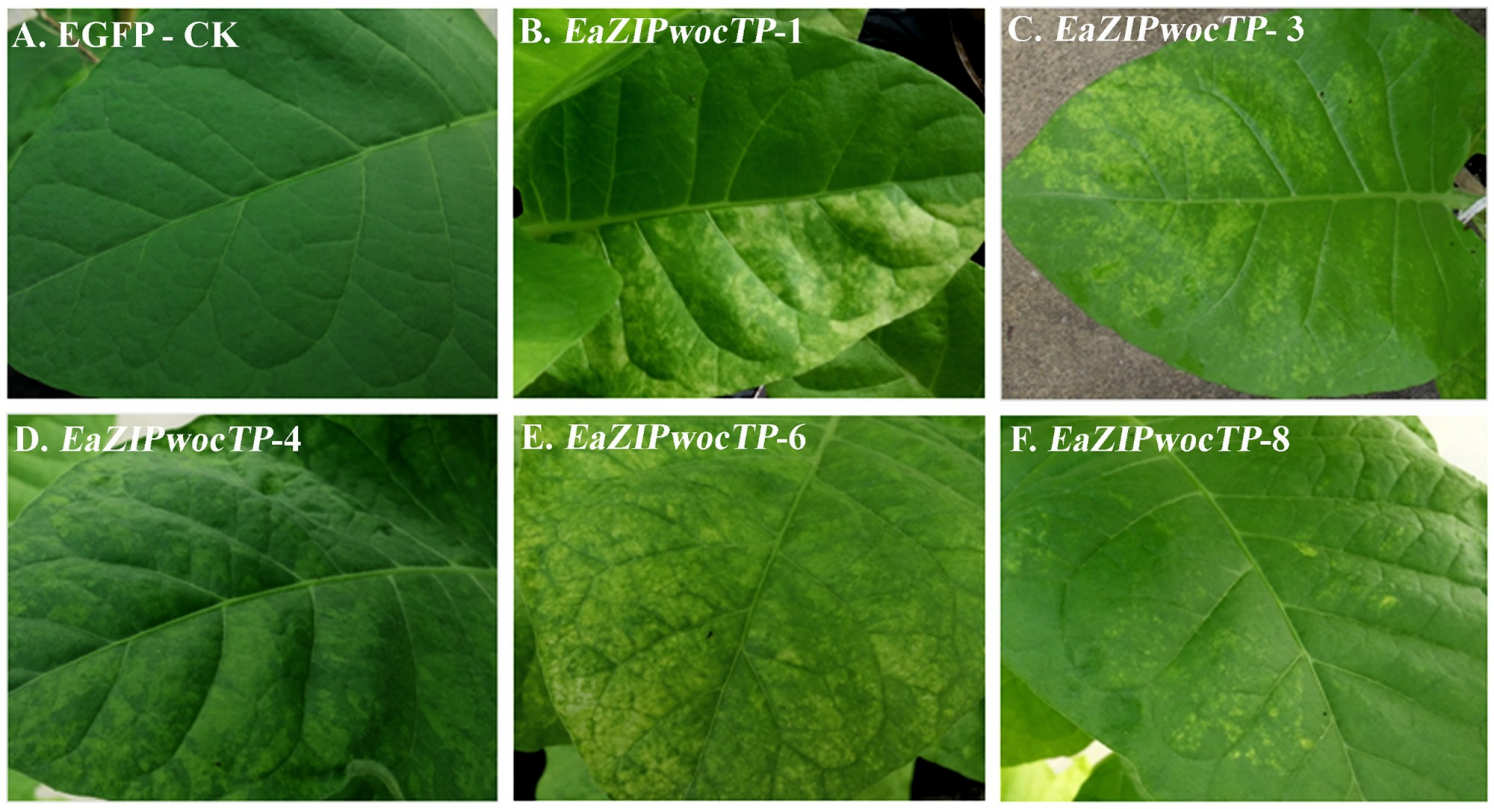
Transgenic tobacco leaves. A. EGFP-CK, plants harboring EGFP gene as a control, plants did not exhibit leaf variegation; B-F. Plants harboring *EaZIPwocTP* gene, which showed different degrees of leaf variegation.

All plants overexpressing the *EaZIPwcTP* and *EGFP* genes did not show any phenotypic abnormality (Figs 5A and 5B and 6A). However, plants overexpressing *EaZIPwocTP* exhibited variegated leaves (Fig 5C). The variegation was conspicuous patches of discoloration without specific patterns (Fig 6). Plants with such variegations did not show other growth abnormalities such as curled leaf structure and thickening of leaf lamina that are typically found in virus-infected plants. The degree of variegation varied among different plant transgenic lines. Some showed variegated patches on only half of leaf throughout the whole plant (Fig 6B), others produced different densities of chlorotic patches or spots (Fig 6C–6F). Quite often the variegation phenotype occurred in all leaves regardless of developmental stages (Fig 5C). In addition, unlike typical virus-infected tobacco plants, flower development and seed production were not affected in all examined plant lines.

**Fig 7:**
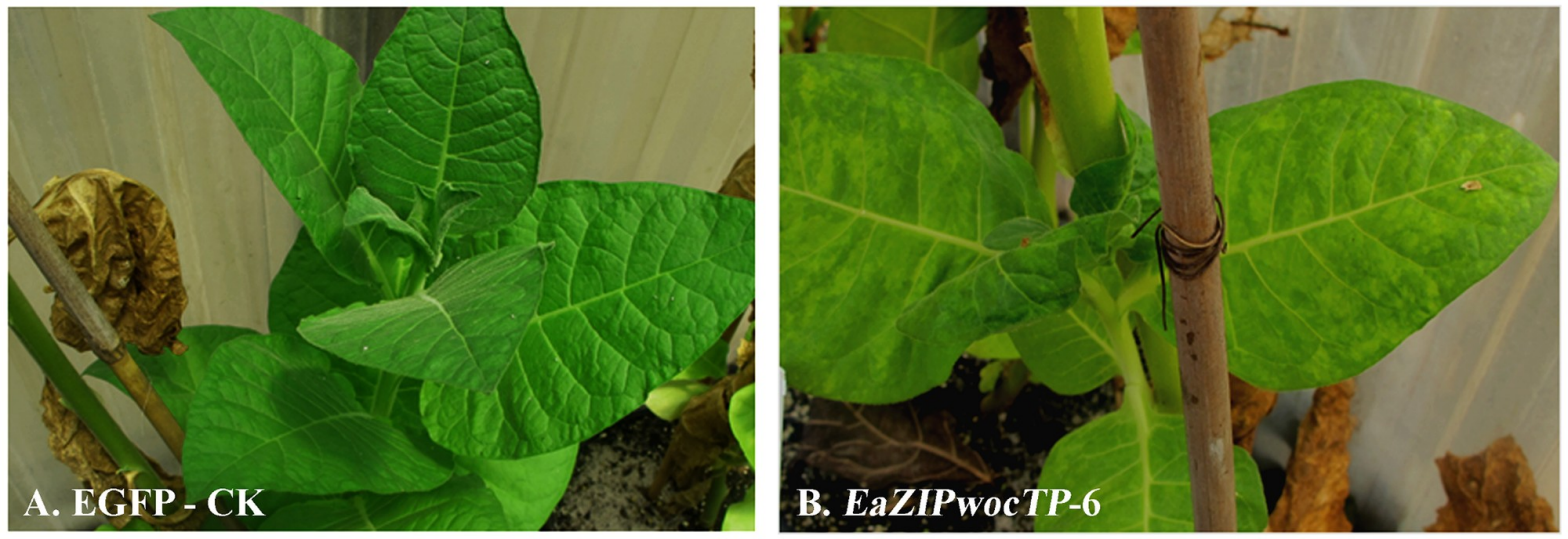
Leaves of tobacco plants derived from axillary shoots. (A) a GFP-CK control plant (no variegated leaves) and (B) an *EaZIPwocTP* line with variegated leaves.

The variegation trait was also faithfully retained in newly produced shoots derived from either axillary buds of mature plants (Fig 7A) or *in vitro* shoot regeneration by culturing excised leaf explants under kanamycin selection (data not shown). The persistence of the variegation (plants propagated from seed germination, axillary buds or regenerated from leaf explants) suggests that the observed leaf phenotypes are associated with the introduced *EaZIPwocTP* in these plants.

### Chloroplast density analysis

**Fig 8:**
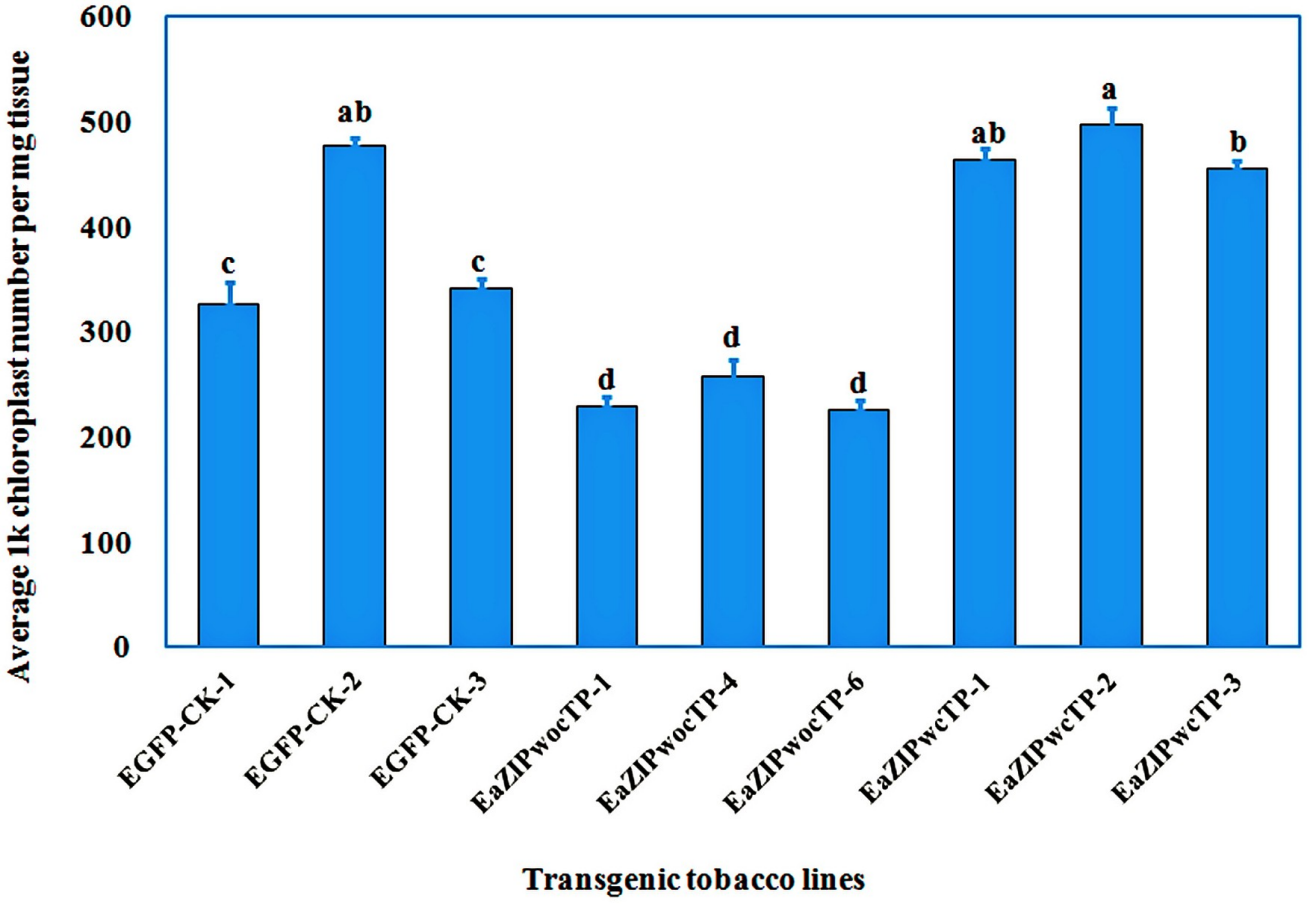
Comparison of chloroplast density of leaf tissues among transgenic tobacco plant lines overexpressing *EGFP* (EGFP-CK), *EaZIPwocTP*, and *EaZIPwcTP*. Samples from three each of *EGFP*, *EaZIPwocTP*, and *EaZIPwcTP* plant lines were examined. Bar represent average 1000 x chloroplasts per mg tissue. Standard errors were indicated (n = 3). Different letters above bars indicate significant difference (*P* < 0.05) among examined leaves according to Fisher’s LSD test.

Variegated leaves of transgenic plants appeared to be more pervious to light than green leaves of EGFP-control plants and *EaZIPwcTP* plants under microscope observation. To determine if such permeable property of variegated leaves is related to a lowered chloroplast density, leaf tissues were collected from transgenic plants containing *EGFP*, *EaZIPwocTP*, and *EaZIPwcTP* were subject to quantification of relative chloroplast density using excised leaf sectors. Results indicate that green leaves from *EGFP* and *EaZIPwcTP* plants contained an average density of 3.82 x 10^5^ and 4.72 x 10^5^ chloroplasts per mg tissue, respectively whereas only 2.38 x 10^5^ chloroplasts per mg tissue were found in variegated leaves overexpressing the *EaZIP* short gene (Fig 8). In other words, the overexpression of the *EaZIPwocTP* resulted in a 37.8% and 49.6% reduction in chloroplast density compared to EGFP transgenic control and *EaZIPwcTP* plants.

## Discussion

Leaf variegation is one of most sought-after traits in the ornamental plant industry as it can dramatically enhance attractiveness and aesthetic quality of plants. Creating novel variegated phenotypes in consumer-favored species or varieties remains a high priority in ornamental breeding efforts. As more knowledge about the underlying mechanisms is revealed through extensive molecular studies, it will become easier to engineer novel variegation by predictable gene transfer approaches. In this study, we demonstrated that the variegation phenotypes were created in tobacco by overexpressing an *EaZIPwocTP* gene. The highly random pattern of variegation is different from the vein-associated variegation reported previously using *Arabidopsis* CHL27 gene antisense construct [31].

*EaZIP* was shown to be a homolog of Mg-protoporphyrin IX monomethyl ester (MPE) cyclase gene found in other species [27]. This enzyme functions to catalyze the formation of divinyl protochlorophyllide from MPE, a critical intermediate molecule for chlorophyll biogenesis inside the chloroplast [27,29,32]. Since the enzyme is encoded by a nuclear gene, it generally necessitates a cTP for the pre-protein or protein precursor to be imported into the final organelle destination. The translocation process is governed by a highly regulated import pathway involving the participation of a variety of specialized protein factors that recognize three domains of cTP [35].

Our alignment analysis in which ZIP peptides from orthologous genes of a variety of plant species were included indicates that the previously amplified *EaZIP* cDNA by 5’-RACE lacks a sequence region encoding a recognizable cTP. The result was unexpected, and it was unknown why the *EaZIP* cDNA isolated from ‘Golden Pothos’ has no cTP. Subsequently, we questioned if overexpressing *EaZIP* devoid of cTP could affect chlorophyll biosynthesis and probably chloroplast development and produce variegated leaves. Thus, transgenic tobacco plants containing both *EaZIP* with or without cTP region were generated. Transgene integration was evidenced by a series of extensive PCR and copy number analyses. The expression of both marker gene and *EaZIP* genes was confirmed by GFP observation and transcript detection with results in agreement with previous findings using similar promoter construct [38]. Only overexpression of the *EaZIPwocTP*, not *EaZIPwcTP*, resulted in leaf variegation. The main difference between these two versions of *EaZIP* is the absence of cTP region in the former and the presence it in the latter. Although cTP is important for translocation of nuclear encoded proteins to chloroplast, previous studies revealed that peptides with mutated or deleted cTP could also be transported into chloroplast but with much reduced efficiency and extended time duration [43,44]. However, proteins imported into chloroplast mediated by mutated cTPs showed up with many aberrations [43]. Should it be, as mentioned above, a true prediction that the first 6 residues of the cloned *EaZIP* cDNA constitute a reduced cTP, the protein import into chloroplast might behave as being guided by a mutated cTP. These aberrant proteins might interfere with cyclase reaction by competitive substrate competition with the endogenous tobacco ZIP proteins. Failure of such reaction can hamper the otherwise normal conversion of divinyl protochlorophyllide from MPE, block chlorophyll biosynthesis and induce accumulation of oxidative MPE that is capable of damaging chloroplasts. Consequently, all these events could result in formation of variegated leaf sectors. The interference of indigenous tobacco ZIP by the abundant aberrant EaZIP proteins depends on the expression level/threshold of the latter which is controlled by a viral promoter. The random pattern of variegation thus can be viewed as a consequence of the fluctuated activities of the viral promoter in different cells/leaf sectors.

Another possible mechanism for leaf variegation in *EaZIPwocTP* could be RNA interference. If transcripts resulted from overexpressed *EaZIPwocTP* could not be properly translated in tobacco, they may interfere the stability of tobacco endogenous *ZIP* transcripts since deduced amino acid sequence of *EaZIPwocTP* shared an 86% identity to the tobacco ZIP [27]. A study of CHL27 using antisense construct resulted in the vein-associated variegation in *Arabidopsis* [31], indicating the possible involvement of RNA interference.

It remains to be determined whether the *EaZIP* short gene will also be able to induce variegation in donor green pothos plants or any other ornamental plant species. Should that be the case, interpretation of variegated phenotype by reduced expression level of *EaZIP* [27] can be further expanded by this study. Nevertheless, the discovery of variegation induction by overexpression of an *EaZIPwocTP* will facilitate the application of biotechnological tools and gene transfer approach in creation of novel aesthetic traits in ornamental plants.

## Supporting information

S1 Fig Amino acid sequence alignment of ZIP peptides from various species. All entries were retrieved from GenBank. Red line indicates putative chloroplast transit peptide cleavage site.
(PDF)
