# Supplementary material for: Overexpression of an EaZIP gene devoid of transit peptide sequence induced leaf variegation in tobacco
Source: PLoS One. 2017 Apr 19;12(4):e0175995. doi: 10.1371/journal.pone.0175995 (PMC5397061; doi:10.1371/journal.pone.0175995)

Overexpression of an *EaZIP* Gene devoid of Transit Peptide Sequence Induced Leaf Variegation in Tobacco

Xiayu Guan[1,2], Zhijian Li[2], Zhiliang Zhang[2,3], Xiangying Wei[1,2], Jiahua, Xie[4], Jianjun Chen[1,2*], and Qingxi Chen[1*]

Supporting information: S1 Fig.

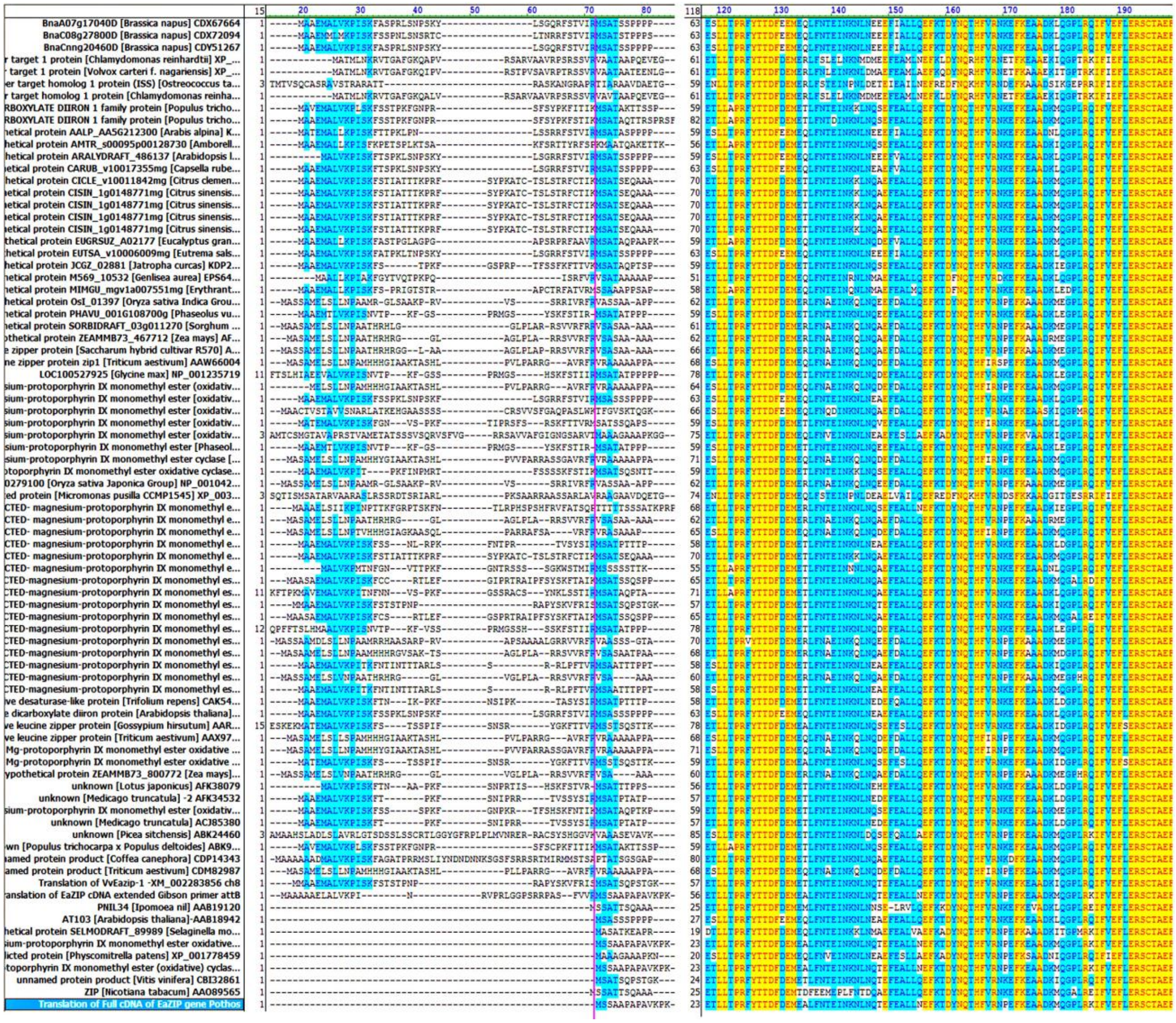

Supplement: S1 Fig — All entries were retrieved from GenBank. Red line indicates putative chloroplast transit peptide cleavage site. (PDF) [file pone.0175995.s001.pdf]
